# Calliphoridae and Mesembrinellidae (Insect: Diptera) Across Different Environments of Rio de Janeiro, Brazil: Synanthropy and Potential Bioindicators, with Notes on Bait Preference

**DOI:** 10.3390/life15121818

**Published:** 2025-11-27

**Authors:** Wellington Thadeu de Alcantara Azevedo, Mariana dos Passos Nunes, Tomaz da Silva Telles Machado, Valmíria Moura Leôncio Albuquerque, Cláudia Soares Santos Lessa, Jeronimo Alencar, Valéria Magalhães Aguiar

**Affiliations:** 1 Department of Microbiology and Parasitology, Biomedical Institute, Federal University of the State of Rio de Janeiro, Rua Frei Caneca, 94, Centro, Rio de Janeiro CEP 20211-040, Brazil; wellingtontaa@yahoo.com.br (W.T.d.A.A.);; 2 Graduate Program in Animal Biology, Federal Rural University of Rio de Janeiro, BR 465, Km 07, Seropédica, Rio de Janeiro CEP 23890-000, Brazil; 3 Graduate Program in Biological Sciences, Federal University of the State of Rio de Janeiro, Av. Pasteur 458, Urca, Rio de Janeiro CEP 22290-240, Brazil; 4 Diptera Laboratory, Oswaldo Cruz Foundation, Rio de Janeiro CEP 21040-360, Brazil

**Keywords:** anthropization, environmental entomology, biomonitors, conservation, forensic entomology, medical entomology

## Abstract

The Atlantic Forest is a biome of great diversity under constant anthropic pressure. This study was conducted in three environments in the state of Rio de Janeiro: rural environment, Seropédica campus (UFRRJ); urban environment, Urca campus (UNIRIO); and forest environment, Três Picos State Park, Cachoeiras de Macacu. We aimed to evaluate the attractiveness of the fauna of Calliphoridae and Mesembrinellidae by two stages of bait decomposition, study their synantropy, and identify bioindicators for each environment. Four traps were installed at each environment, two containing preserved beef liver and two containing beef liver with 48 h of putrefaction. Samples were collected quarterly, between June 2021 and May 2023. A total of 5476 dipterans were collected, with five Calliphoridae species (77.1%) and 11 Mesembrinellidae (22.9%). *Laneella nigripes* showed a preference for liver baits with 48 h of putrefaction. Mesembrinellidae species, *Hemilucilia benoisti* and *Paralucilia nigrofacialis* were asynanthropic, occurring exclusively in the forest environment. *Hemilucilia segmentaria* and *H*. *semidiaphana* were also asynanthropic, but occurred in urban and/or rural environments. *Chrysomya* and *Cochliomyia* genera and *Lucilia cuprina* were synanthropes. Eight potentially bioindicator species were identified for the forest environment and four for rural environments.

## 1. Introduction

The Atlantic Forest is composed of various types of vegetation, including forests, sandbanks, mangroves, and high-altitude fields. Considered one of the world’s 36 biodiversity hotspots, this biome is home to more than 15,000 endemic vascular plant species, although it occupies only 7% of its original area [1,2]. In addition to its rich flora, the Atlantic Forest has one of the most diverse fauna on the planet, with a high number of endemic species. The biome extends across 17 Brazilian states and has been under constant anthropogenic pressure due to resource extraction and intense urbanization [3,4,5,6].

The loss of biodiversity due to human activity is one of the main reasons for establishing environmental protection areas, and has been the subject of extensive debate [7,8,9]. As a result, the number of environmental protection areas in fragments of the Atlantic Forest has grown in recent years [5], although, due to its high biodiversity and complex biotic interactions, it should be an even greater focus of concern [7,10].

In this context, the class Insecta, the most diverse animal group with three times more species than all other groups combined, stands out for its great ecological importance. Insects act as pollinators, decomposers, vectors of pathogens, parasites, and economic pests [11,12]. Additionally, they are excellent environmental bioindicators due to their small size, sensitivity to changes, short life cycle, and high abundance [7]. Thus, population analyses of entomofauna can provide valuable information on the state of preservation or degradation of an environment, whether due to natural or anthropogenic factors [4,13].

Diptera is one of the most diverse orders of insects and is present in almost all environments and niches [14,15,16,17]. The family Calliphoridae, popularly known as blowflies [18], is widely distributed worldwide and consists of more than 1000 species with about 150 recognized genera [19]. Larvae of some species in this family can be biontophagous (feeding on living organic matter, such as healthy tissue from a host), necrophagous (feeding on decomposing organic matter, such as garbage, feces, carcasses), or necrobiontophagous (feeding on necrotic tissue in living hosts) [20], and can cause obligate and facultative myiasis, which makes these dipterans extremely important in animal [21] and human [22] health. In forensic entomology, data on their biology has also been used to solve criminal cases, such as identifying suspects through molecular analysis, transposition of corpses, and, mainly, estimating the post-mortem interval [23,24,25].

The family Mesembrinellidae, previously considered a subfamily of Calliphoridae [26], shares these necrophagous habits and comprises a small group of exclusively neotropical flies, highly related to forest areas, where they are abundant and diverse. Therefore, they are considered potential biological indicators for preserved forest areas, responding to different types of environmental impacts [4,24,25,26,27].

The forest species of Calliphoridae and Mesembrinellidae, however, are poorly known. This is largely due to the difficulty of breeding or even keeping these insects outside their habitat for long enough, making it difficult to observe their biological characteristics, such as reproduction, vagility, and longevity, and ecological characteristics [4,7,27]. In addition, even the taxonomic classification of these dipterans is still the subject of extensive discussion, and Mesembrinellidae has been elevated to family status, no longer being a subfamily of Calliphoridae [26,28].

The concept of synanthropy is used for species, both native and exotic, that benefit from anthropized environments, either directly or indirectly, with the exception of domesticated animals. The benefits include increased population density and reproductive capacity and/or survival. Species that respond negatively to anthropization are called asynanthropic [29]. An anthropized environment is understood to be any environment that has undergone changes due to human action, whether through the construction of cities and industrial areas, or through the implementation of basic sanitation, agricultural, or agro-industrial activities [30]. As a consequence, the natural environment becomes fragmented, amplifying edge effects, which results in population changes in the marginal regions of the remnants. Other important changes are wind, light, humidity, and temperature. The effects of these changes are evident up to 500 m inside the remnants [4].

Studies in fragments of Atlantic Forest in the State of Rio de Janeiro—Tijuca National Park [24,25] and Tinguá Biological Reserve [4,27], among others—have demonstrated a great diversity of species from the Calliphoridae and Mesembrinellidae families, which are characteristically synanthropic, rarely recording highly synanthropic species [24].

Knowledge of the Calliphoridae and Mesembrinellidae dipteran fauna in this Atlantic Forest unit will enable us to understand the diversity of species in these families and, through faunal parameters, identify bioindicator species and the degree of conservation of the environment in question. In addition, understanding the responses of these dipterans to the abiotic factors that affect their distribution may contribute to enriching knowledge about the behavior of the species captured in this environment.

Physical measurements of environmental variables, although accurate, require interpretation efforts under a set of factors that may be acting in synergy on the environment. Therefore, their interpretation depends on laboratory studies under specific conditions and is rarely extrapolated to natural conditions [31]. Bioindicators, in turn, are organisms, populations, or communities that exhibit measurable responses to certain changes, the interpretation of which allows us to indicate the health of the ecosystem. Their most common application is in environmental quality studies, measuring impacts caused mainly by anthropogenic activities, and to identify biogeographical changes [31,32,33,34]. Insects are widely used as bioindicators because they meet the main selection criteria: high abundance and specific reactions to environmental changes [33].

The application of bioindicators in studies over a given space and time is called biomonitoring, allowing the quantification of changes observed in bioindicators. Compared to other methods, this one benefits from the relationship between the organism and its environment, allowing the evaluation of cumulative impacts and the establishment of safety thresholds for environmental impacts [31,34].

The objective was to study the synanthropy of the recorded species, identifying possible bioindicators based on their the degree of preference for three environments using two types of baits in the State of Rio de Janeiro: (a) forest area—Três Picos State Park (RJ); (b) rural area—cattle farming on the campus of the Federal Rural University of Rio de Janeiro; and (c) urban area—campus of the Federal University of the State of Rio de Janeiro, in the Urca neighborhood; and to apply this knowledge in determining bioindicators that can infer the degree of preservation of forest areas through the assessment of anthropic influence.

## 2. Materials and Methods

**Figure 1:**
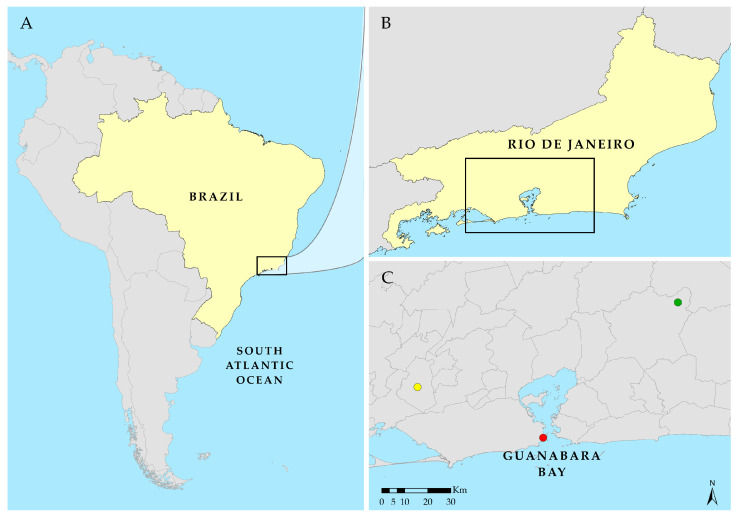
Map of study areas: (**A**) South America, Brazil; (**B**) State of Rio de Janeiro; (**C**) sampling sites, highlighting the three collection sites for Calliphoridae and Mesembrinellidae studied in the state of Rio de Janeiro, Brazil: Forest (●), in a primary forest zone of the Três Picos State Park, municipality of Cachoeiras de Macacu; Rural (●), UFRRJ campus, municipality of Seropédica; and urban (●), UNIRIO Urca campus, in the municipality of Rio de Janeiro. Source: Designed by the authors using ArcGis 10.7.1. Shapefiles were obtained from IBGE: https://www.ibge.gov.br/geociencias/cartas-e-mapas/bases-cartograficas-continuas.html, accessed on 6 November 2025.

The collections were carried out in three distinct areas of the state of Rio de Janeiro, in locations with forest, rural, and urban characteristics (Figure 1).

### 2.1. Forest Area—Três Picos State Park (PETP)

Samples from the forest environment were collected in the vicinity of the PETP headquarters located in the municipality of Cachoeiras de Macacu.

PETP was created in 2002 with the aim of preserving remnants of the Atlantic Forest and recovering degraded areas in the Serra do Mar mountain range, as well as promoting environmental education. In 2009, its jurisdiction was expanded, currently encompassing approximately 58,790 ha, covering territories in the following municipalities: Cachoeiras de Macacu, Nova Friburgo, Teresópolis, Guapimirim, and Silva Jardim. It is currently the largest fully protected area in the state, comprising the central ecological corridor of the Atlantic Forest in Rio de Janeiro. The great diversity of habitats included in this conservation unit and its altitude gradient, which varies from 100 to 2310 m, are reflected in the diversity of fauna and flora contained in the park, which is considered by experts to be one of the priority areas for the conservation of the Atlantic Forest in Brazil [2].

The PETP serves not only as an environmental protection area, but also as an ecological corridor between several conservation units. Among them are the Serra dos Órgãos National Park and the APAs of Petrópolis, Macaé de Cima, and Rio São João. However, in addition to the areas open to visitors, the park faces anthropic pressure from private properties for expropriation, irregular occupations, hunting, plant extraction, and forest fires [2].

### 2.2. Rural Area—Federal Rural University of Rio de Janeiro (UFRRJ)

The representative rural location was found within the Seropédica campus of UFRRJ, approximately 119 km from the forest area. UFRRJ was originally founded as the Higher School of Agriculture and Veterinary Medicine in 1910, in the Maracanã neighborhood, and was only transferred to Seropédica in 1948. After several name and location changes, it was officially renamed UFRRJ in 1967. Currently, UFRRJ consists of four campuses (Seropédica, Nova Iguaçu, Três Rios, and Campos dos Goytacazes) [35].

The area was selected due to the abundance of rural animals, with cattle, poultry, horses, and dogs, and low population density, together with the characteristics of the location, with no dense vegetation and extensive grass fields. In addition, this area has little residential waste production and no industrial waste, and low y traffic of people and vehicles, but it has an abundance of organic matter of animal origin, mainly from excrement.

### 2.3. Urban Area—Federal University of the State of Rio de Janeiro (UNIRIO)

The urban area studied was located within the UNIRIO Institute of Biosciences, in the Urca neighborhood of Rio de Janeiro, approximately 81.5 km from the forest area. It was created in 1969, with the Federation of Isolated Schools of the State of Guanabara. In 1975, with the merger of the states of Guanabara and Rio de Janeiro, it became known as the University of Rio de Janeiro, already represented by the acronym UNIRIO, with its current name only being defined in 2003. It currently offers 45 undergraduate and 54 graduate courses, distributed across five campuses (Botafogo, Tijuca, Centro, and two in the Urca neighborhood) [36].

These locations were chosen due to their high population density, circulation of people and vehicles, and waste production, together with the presence of fauna typical of urban environments (pigeons, rats, cockroaches, among others) and the absence of dense vegetation or grassy fields.

### 2.4. Sampling Method

Collections were carried out quarterly, between June 2021 and May 2023, with four traps exposed per point for a period of 48 h in each collection. The traps followed the description by Mello et al. [37], consisting of a PVC tube base with side holes for insects to enter, inside which odor-attractive bait is placed, and a transparent polyethylene container that fits over the base, containing a funnel to retain insects through positive phototropism. Two types of bait were used: two traps containing preserved beef liver; and two traps containing beef liver decomposed in a sterile environment for 48 h. After collection, the specimens were transferred to sequentially numbered polyethylene containers and recorded on field cards with a description of the sampling point and date. The collected individuals were then sacrificed using cotton soaked in a solution of ethyl alcohol and ethyl acetate. These containers containing the specimens were transported to the Diptera Studies Laboratory of the Federal University of the State of Rio de Janeiro (LED-UNIRIO), where they were kept in a freezer at −5 °C until processed.

The processing of the material consisted of a screening based on morphological characteristics to separate the Calliphoridae and Mesembrinellidae dipterans from other possible species collected in the trap. For taxonomic identification, the insects were dried under incident light on absorbent paper. The insects were pinned, and species identification was performed based on direct observation of morphological characters visible under a stereoscopic microscope and consultation of the respective descriptions/diagnoses of the species, using dichotomous keys developed by Mello [38] and Kosmann et al. [39]. Part of the samples (n = 4 or less, when available) were pinned to be added to the entomological collections of the LED and the National Museum of the Federal University of Rio de Janeiro (UFRJ), and the rest of the material was stored in entomological envelopes with labels containing collection and identification information and kept in the LED collection.

### 2.5. Synanthropy Study

For the synanthropy study, the captures were added up, considering the total for each environment: forest, rural, and urban areas. The Sorensen’s similarity index was used to compare the environments. Synanthropic indices (SI) were calculated according to Nuorteva [40], using the following formula:

$$IS=\frac{2a+b-2c}{2}$$

where

a = percentage of individuals of a given species collected in the urban area;
b = percentage of individuals of a given species collected in the rural area;
c = percentage of individuals of a given species collected in the wilderness area.

### 2.6. Bioindicator Analysis

For the analysis of bioindicators, the same methodology used for the study of synanthropy was adopted. To determine the bioindicator species, the multipatt function of the indicspecies package was used with 999 permutations. Statistical analyses were performed in R Studio 2025.05.1, assuming a significance level of 5% (*p* = 0.05).

### 2.7. Bait Preference

After verifying the normality of the data, hypothesis tests were used to evaluate the preference of the most abundant species for the two stages of bait decomposition, preserved and 48 h of putrefaction. Statistical analyses were performed in R Studio, assuming a significance level of 5% (*p* = 0.05).

## 3. Results

During the two years of study, 4202 dipterans were collected, totaling 21 species of the Calliphoridae and Mesembrinellidae families (Table 1). Of this total, 86.9% were representatives of five species of the Calliphoridae family, with *Lucilia eximia* being the most abundant species (60.1% of the total collected). The Mesembrinellidae family represented 13.1% of this dipterofauna, contributing with the occurrence of ten species, with *Laneella nigripes* being the most abundant species (7.0% of the total).

### 3.1. Bait Preference

Comparing the preference of Calliphoridae and Mesembrinellidae for the two stages of bait decomposition analyzed, there is an apparent preference of some species for one stage. The abundance of species in the two stages of bait decomposition shows greater capture in bait with 48 h of putrefaction (2463 individuals) compared to fresh bait (1739 individuals) (Table 1).

**Figure 2:**
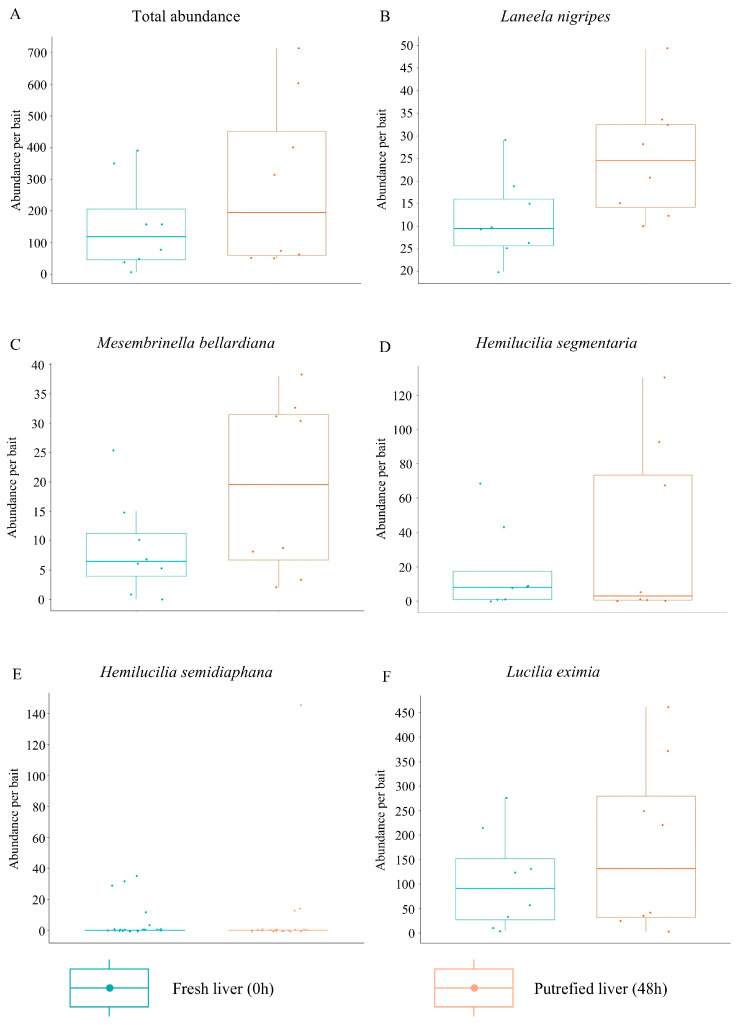
Boxplot of total abundance (**A**) and the most abundant species (n > 200) of Mesembrinellidae (**B**—*Laneela nigripes*; and **C**—*Mesembrinella bellardiana*) and Calliphoridae (**D**—*Hemilucilia segmentaria*; **E**—*Hemilucilia semidiaphana*; and **F**—*Lucilia eximia*) captured at different stages of decomposition of bovine liver (preserved and with 48 h of putrefaction) in three environments in the state of Rio de Janeiro: Forest: Jequitibá Nucleus of the Três Picos State Park, municipality of Cachoeiras de Macacu; Rural: Cattle Farming at the Federal Rural University of Rio de Janeiro, municipality of Seropédica; and Urban: Institute of Biosciences at the Federal University of the State of Rio de Janeiro, Urca Campus, municipality of Rio de Janeiro, between August 2021 and May 2023.

After verifying the normality of the data using the Shapiro–Wilk test (S-W = 0.691, *p*-value < 0.001 for preserved liver, W = 0.558, *p*-value < 0.001 for putrefied liver), the Wilcoxon test showed that there was no difference between the attractiveness of the baits (W = 284.5, *p*-value = 0.951; Figure 2A). Analyzing the preference of the most abundant species of each family (n > 200), it was possible to observe that *La. nigripes* showed a preference for liver with 48 h of putrefaction (t = −2.373, gl = 12.461, *p*-value = 0.035), while the species *Mesembrinella bellardiana* (t = −1.755, df = 10.783, *p*-value = 0.108), *Hemilucilia segmentaria* (W = 296.5, *p*-value = 0.861), *Hemilucilia semidiaphana* (W = 311, *p*-value = 0.475), and *L. eximia* (W = 295.5, *p*-value = 0.885) showed no preference (Figure 2B–F).

### 3.2. Study of Synanthropy

Analyzing the three areas studied, 21 species were captured in the three environments, 10 of Calliphoridae and 11 of Mesembrinellidae. The Sorensen similarity observed between the three environments was low (Table 2), with the rural and urban environments being the most similar to each other (S = 0.667), with four species in common, while the rural and forest environments were the most distinct (S = 0.174), with two species in common. The forest and urban environments also showed low similarity (S = 0.286), with three species in common between the two environments.

It can be observed that some species occurred in greater abundance in certain environments (Table 3). The 11 Mesembrinellidae species occurred exclusively in the forest environment. Despite low capture rates in rural and urban environments, it can be noted that the genera *Chrysomya* Robineau-Desvoidy, 1830, and *Cochliomyia* Townsend, 1915, showed a preference for the rural environment, occurring in greater abundance, and in some cases exclusively in this environment. *Hemilucilia segmentaria* was more abundant in forest and urban environments, while *H. semidiaphana* occurred in greater abundance in the forest environment, with no records in the rural environment. *L. eximia* occurred in greater abundance in the forest environment, but *Lucilia cuprina* was recorded only in the rural environment. Five species of Calliphoridae occurred in the forest area, seven in the rural area, and five in the urban area.

**Figure 3:**
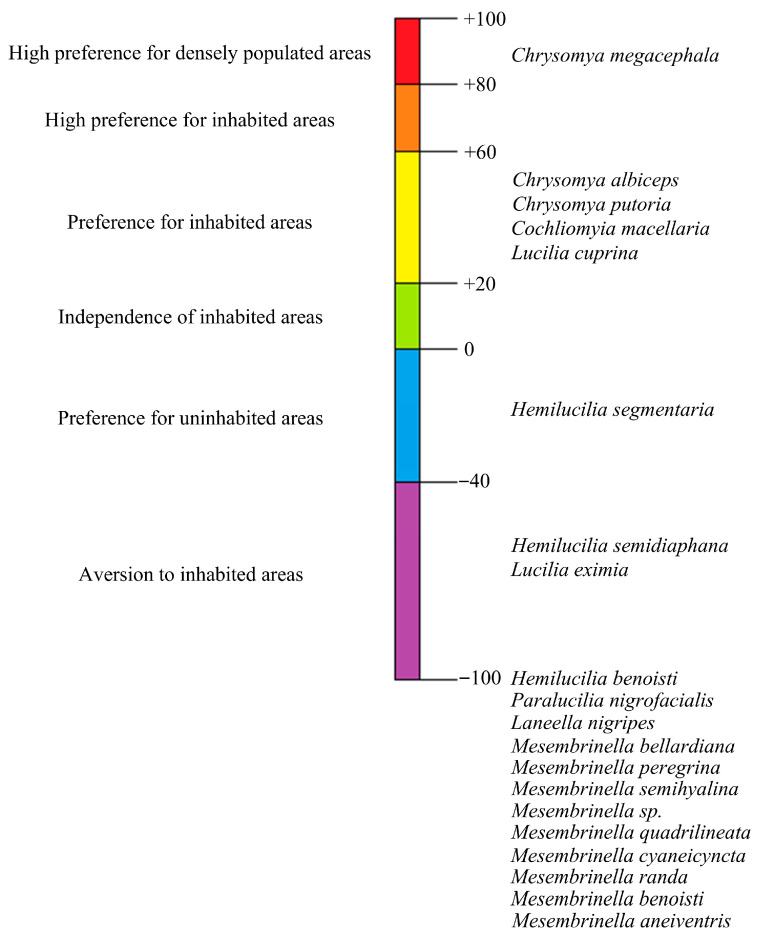
Synanthropic classification of species of the Calliphoridae and Mesembrinellidae families captured at different stages of decomposition of bovine liver (preserved and with 48 h of putrefaction) in three environments in the state of Rio de Janeiro: Forest: Jequitibá Nucleus of the Três Picos State Park, municipality of Cachoeiras de Macacu; Rural: Cattle farm of the Federal Rural University of Rio de Janeiro, municipality of Seropédica; and Urban: Institute of Biosciences of the Federal University of the State of Rio de Janeiro, Urca Campus, municipality of Rio de Janeiro, between August 2021 and May 2023.

Through the synanthropy index, it was found that all species of the Mesembrinellidae family showed total aversion to inhabited environments, being considered asynanthropic (I.S. = −100) (Figure 3). The synanthropy of the Calliphoridae species varied, with *Chrysomya megacephala*, *Chrysomya albiceps*, *Chrysomya putoria*, *Cochliomyia macellaria*, and *L. cuprina* proving to be synanthropic (I.S. between 20 and 100), while *H. segmentaria*, *H. semidiaphana*, *L. eximia*, *Hemilucilia benoisti*, and *Paralucilia nigrofacialis* occurred as asynanthropic (I.S. between 0 and −100). There were no hemi-synanthropic species (I.S. between 0 and 20).

### 3.3. Bioindicator Analysis

The bioindicator analysis selected 12 species with potential application, as they are strongly associated with the forest or rural environment (Table 4). In contrast, no species proved suitable for use as a bioindicator or biomonitor in urban environments. For the forest environment, the most significant species were *La. nigripes*, *M. bellardiana*, and *L. eximia* (*p* = 0.001). For the rural environment, the most significant species were *Co. macellaria* (*p* = 0.001) and *L. cuprina* (*p* = 0.002).

## 4. Discussion

Mesembrinellidae are exclusively forest-dwelling and neotropical, with *M. bellardiana* and *La. nigripes* often reported as abundant in well-preserved forest areas. Gadelha et al. [4,27] reported the capture of 10 species of this family, eight of which occurred in the present study, when studying a 2000 m edge gradient of the Tinguá Biological Reserve, Rio de Janeiro, using sardines as bait. In studies in Tijuca National Park using rat carcasses as bait, Carvalho et al. [24] and Azevedo et al. [25] found three species of the Mesembrinellidae family that were also present in Três Picos State Park, as well as the occurrence of *L. eximia*, *H. segmentaria*, and *H. semidiaphana.* The differences in richness may have occurred due to the baits used. Sardine bait, due to its strong odor, is very effective in capturing necrophagous dipterans, allowing for a higher richness to be captured. Rat carcass bait, as well as the liver used in this study, has a milder odor. In addition, studies indicate that the use of a whole organism, due to the presence of all organs, may be more attractive depending on the preferences of each species due to the release of volatile organic compounds from different sources [41,42,43]. Of particular note is the occurrence of *Mesembrinella currani*, whose known distribution is expanded by this study, since its records were previously restricted to the North and Northeast regions and other countries further north of Brazil [44].

As the environments studied become more anthropized, only a few species of the Mesembrinellidae family are able to survive. Species of the Calliphoridae family, on the other hand, thrive in this type of environment, maintaining their abundance and richness. When studying a fragment of Atlantic Forest in Recife, Brazil, Carmo et al. [42] portray this transition by finding only two species of the Mesembrinellidae family, *M. bellardiana* and *Mesembrinella bicolor* (Fabricius, 1805), in low abundance, while species of the Calliphoridae family occurred in high abundance. This study also highlights the replacement of species from the Calliphoridae families. Calliphoridae of the genus *Hemilucilia*, as well as *L. eximia*, are commonly reported in the literature as abundant in forest environments, with their abundance decreasing as the transition to a drier and more open environment occurs, where species of the genus *Cochliomyia* begin to dominate the environment.

When studying a preserved fragment of Caatinga in the municipality of Serra Talhada, in the state of Pernambuco, Vasconcelos et al. [45] observed the dominance of *Co. macellaria*, followed by *C. albiceps*. These results highlight the *turnover* observed in anthropized areas, when species of the genus *Cochliomyia*, native to the Americas, begin to gain an advantage, and especially species of the genus *Chrysomya*, which were introduced in the 1970s and can now be considered cosmopolitan species [46], which, since then, have increasingly restricted the distribution of the genus *Cochliomyia* because they are more voracious in the larval stage [28,47].

The presence of *M. bellardiana* in forest remnants, despite the marked presence of Calliphoridae, is common in the literature, showing that this species has great plasticity [41,48,49].

The analysis of dipterofauna between forest, rural, and urban environments revealed, for the most part, species typical of each environment, as widely reported in the literature. The dipterofauna of Calliphoridae and Mesembrinellidae differed predominantly from the fauna recorded in rural and urban environments. In these environments, it was possible to observe that there was no occurrence of species of the Mesembrinellidae family, as well as two species of the Calliphoridae family, *H. benoisti* and *P. nigrofacialis*. The absence of Mesembrinellidae in rural and urban environments was expected, since these species are often associated with well-preserved forest environments [27]. Souza et al. [50], when studying the diversity of Calliphoridae and Mesembrinellidae in Maranhão, reported the occurrence of Mesembrinellidae exclusively in preserved forest environments. This family was also abundantly collected by Amat et al. [51] in the Amazon rainforest.

The occurrence and dominance of species of the genera *Chrysomya* and *Cochliomyia* in urban and rural environments were also expected. The genus *Cochliomyia* comprises the necrophagous dipterofauna of South America and has been affected by the invasion of the genus *Chrysomya* in recent decades [9], which easily occupies new spaces due to its high plasticity.

In this sense, the greater abundance of *L. eximia* in these environments, as well as *H. segmentaria* in the urban environment, was quite atypical. One factor that may have contributed to these results was the restriction on movement imposed by the COVID-19 pandemic, which meant that the sampled environments had less movement of people and vehicles, reducing anthropogenic impacts. In the urban environment, in addition, the proximity to the hills of Babilônia and Urca, which have preserved vegetation, albeit to a lesser extent, may have allowed populations from these locations, due to the favorable conditions caused by the pandemic, to move to the collection site, while the species more typically found in urban environments were not as present because they did not have the resources typically available under normal conditions [44].

Urbanization can have different effects on biodiversity. On the one hand, it can homogenize the environment and reduce the availability of niches, favoring the dominance of species that are better adapted to this environment (usually exotic), which can even lead to the extinction or displacement of other species [52]. On the other hand, it can increase biodiversity by incorporating exotic species or by offering a greater diversity of resources and niches [8]. Studies in anthropized regions such as the BioParque do Rio de Janeiro (formerly RioZoo) [53] reveal an abundance of some species of the Calliphoridae family that are characteristically synanthropic and the absence of assinanthropic species. In transitional environments, such as the Rio de Janeiro Botanical Garden, a gradient in community structure was observed towards the interior of the forest, with a drastic decrease in the capture of synanthropic species and the emergence of highly assinanthropic species [13]. Gadelha et al. [4], in a study at the Tinguá Biological Reserve in the state of Rio de Janeiro, studied the edge effect by sampling different gradients of the park, finding the dominance of *C. megacephala* at the edge, among other characteristically urban Calliphoridae, and its reduction in the innermost points, while the more forest-dwelling species of Calliphoridae and Mesembrinellidae became more abundant. Luz et al. [54], when studying different physiognomies of the city of Niterói, in the state of Rio de Janeiro, found *C. megacephala* to be dominant in sandbank and mangrove environments, where human activity and the presence of waste were striking, and the dominance of *H. semidiaphana* with a marked reduction in the abundance of species of the genus *Chrysomya* in the forest environment, in addition to the occurrence of *M. bellardiana* exclusively in this environment.

The Sorensen index assesses the similarity between two communities, taking into account only the number of species that compose this community [55]. It is closely related to beta diversity, reflecting the degrees of *turnover* and *nestedness* between environments, reflecting the importance of possible evolutionary processes in shaping the current distribution pattern [56]. Unexpectedly, the forest environment showed greater similarity to the urban environment than to the rural environment. As mentioned earlier, this observation may be a consequence of the COVID-19 pandemic period, as well as the proximity to areas of remaining forest.

Therefore, the synanthropy of the species of the Mesembrinellidae family found in this study corroborates the literature, confirming that these are species that occur exclusively in forest environments (I.S. = −100). The synanthropy indices of the species *L. eximia*, *H. segmentaria*, and *H. semidiaphana* vary widely in the literature. For species of the genus *Chrysomya*, synanthropy indices were within the expected range.

The use of physical-chemical measures as indicators of environmental degradation is problematic, as it only records the moment of collection, requiring a large number of samples for efficient temporal monitoring. In addition, the sensitivity of these measurements can be influenced by the distance from the source of the disturbance [9,57]. To overcome this situation, the use of organisms and their biological responses has been widely studied [10,58,59].

When assessing species distribution, it is possible to observe levels of adaptation to environmental changes, noting that different species are distributed in different locations. The Mesembrinellidae family comprises a small group of exclusively neotropical flies, closely associated with forest areas, where they are highly abundant and diverse. They are considered biological indicators for preserved forest areas, responding to different types of environmental impacts [7,24,25,60]. Calliphoridae, on the other hand, have a great capacity to adapt to environments modified by humans, with some species classified as synanthropic [4].

Analyzing the eight species with potential for application as bioindicators/biomonitors of the forest environment identified by the analysis and their mentions in the literature, the species that proved most appropriate for application were *La. nigripes*, *M. bellardiana*, and *L. eximia*. With the exception of *M. bellardiana*, the species have a known life cycle and published laboratory studies, and all have population patterns widely reported in the literature [25,27,61,62]. Thus, it is possible to infer environmental quality by analyzing only the presence/absence and abundance of these species in forest fragments, where their abundance tends to be higher in more preserved environments [4,54], and *La. nigripes* tends to cease to occur in minimally anthropized environments [44].

The rural environment, on the other hand, was related to four species with bioindicator/biomonitor potential. However, evaluating the historical context of the captured species, comparing with reports in the literature and considering how atypical the captures in the urban environment were, it is possible to select the species *Co. macellaria* as a bioindicator/biomonitor for this area. Species of the genus *Chrysomya* are not good indicators, as they are generally very abundant in urban areas [13,54]. Although this pattern was not observed in this study, these would be potentially applicable species for the detection of anthropogenic disturbances, with their occurrence in preserved areas serving as a warning, as they are highly related to anthropized environments [7].

Bioindicators reflect the quality of the environment, allowing us to monitor environmental changes. They often live in specific environments and are vulnerable to sudden changes, such as pollution and other anthropogenic impacts, and climate change. At a specific level, morphological and population changes can be observed, such as a reduction in their abundance. At the ecosystem level, changes in abundance and richness can be observed, mainly [32,63]. However, the application of bioindicators requires some caution. The selection of these organisms depends on several criteria, such as ease of collection and identification, as mentioned above. Morphologically, species in these families differ from the others by having metallic blue or green coloration, although in specimens of the family Mesembrinellidae this characteristic is often restricted to portions of its abdomen, and by the presence of two notopleural bristles. In addition, a row of bristles is present in the meron, a characteristic shared with the Sarcophagidae family. The species in these families differ from each other by the shape of the midrib (M) of their developed wings, which is distinctly angular in the family Calliphoridae and slightly curved in the Mesembrinellidae. In addition, the intensity of sampling can impact the community if too pronounced, while undersampling can provide misleading results [64].

It is also important to note that it is possible to determine the attractiveness preference of dipterans for different types of bait and stages of decomposition. Evidence suggests that baits in an advanced stage of decomposition attract specific groups of Diptera, while preserved bait is more attractive to other species, mainly due to the profile of volatile organic compounds released by each bait [41,42,43]. Moreira et al. [65] reported the preference of Calliphoridae for bovine liver bait in more advanced stages of decomposition. Understanding the attractiveness of dipterans to different stages of bait decomposition aims to provide support for forensic entomology, due to the forensic relevance of this group of insects, enabling the generation of important information to be applied in this area [23,24,25]. Studies indicate that dipterans of the Calliphoridae family show a pattern of succession in relation to the degree of decomposition of the substrate [25,66]. These species are able to identify the substrate shortly after tissue death, even at great distances [38,67]. Some species show a preference for the early stages and others for the more advanced stages of organic matter decomposition, which makes them excellent forensic indicators when it comes to determining the Post-Mortem Interval (PMI) [23].

The ecology of species in the Mesembrinellidae family is less well known. It is not known for certain what the adults of this species feed on, nor the substrate used for larval deposition, with some authors suggesting that they preferentially feed on plant matter and feces [68,69]. However, their capture using organic matter of animal origin has proven to be effective. Among the most abundant species captured, only *La. nigripes* showed a preference among the stages of decomposition of the liver bait, being more abundant in bait that had been rotting for 48 h. It is known, however, that, like Calliphoridae, these species show a pattern of succession in carcass colonization and are considered good indicators for estimating the PMI in forest areas [25,66].

## 5. Conclusions

The most abundant Calliphoridae species are *L. eximia* and *H*. *segmentaria*. The most abundant Mesembrinellidae species were *M. bellardiana* and *La. nigripes*. Some species show variation in relation to the distance from the edge of the forest fragment studied, such as *La. nigripes* and *H. semidiaphana*, while others seem to be more related to the characteristics of each sampling point, such as *L. eximia.* Precipitation, temperature, and relative humidity are also correlated with the abundance of some species, *H. benoisti*, *H. semidiaphana*, *L. eximia*, *P. nigrofacialis*, *M. bellardiana*, and *Mesembrinella peregrina*.

When comparing this fauna with those recorded in rural and urban environments, there is an absence of Mesembrinellidae and two species of the Calliphoridae family, *P. nigrofacialis* and *H. benoisti*, in urban and rural areas, which are classified as synanthropic. In addition, species of the genera *Chrysomya* and *Cochliomyia* and the species *L. cuprina*, considered synanthropic in this study, occur with synanthropy ranging from 50 to 72. The species *L. eximia* and *H. segmentaria* occur in all three environments, with synanthropy of −30.6 and −80.9, respectively, while *H. semidiaphana* does not occur in the rural environment, with synanthropy of −66.8. Based on these results, eight species were defined as having potential for application as bioindicators/biomonitors of the forest environment, notably *La. nigripes*, *M. bellardiana*, and *L. eximia*, and four for the rural environment, notably the species *Co. macellaria.* No bioindicators were found for the urban area. Regarding the stage of decomposition of the beef liver bait used to capture dipterans, only *La. nigripes* shows preference, being more abundant in bait with 48 h of putrefaction.

## Figures and Tables

**Table 1:** Absolute and relative abundance of species of the Calliphoridae and Mesembrinellidae families captured at different stages of decomposition of bovine liver (preserved and with 48 h of putrefaction) in three environments in the state of Rio de Janeiro: Forest: Jequitibá Nucleus of the Três Picos State Park, municipality of Cachoeiras de Macacu; Rural: Cattle farm of the Federal Rural University of Rio de Janeiro, municipality of Seropédica; and Urban: Institute of Biosciences of the Federal University of the State of Rio de Janeiro, Urca Campus, municipality of Rio de Janeiro, between August 2021 and May 2023.

Taxa	0 h		48		Total	
	n	%	n	%	n	%
Calliphoridae						
Luciliinae						
*Lucilia eximia* (Wiedemann, 1819)	1015	58.4	1488	60.4	2524	60.1
*Lucilia cuprina* (Wiedemann, 1830)	12	0.7	8	0.3	20	0.5
Chrysomyinae						
*Chrysomya megacephala* (Fabricius, 1794)	33	1.9	26	1.1	59	1.4
*Chrysomya albiceps* (Wiedemann, 1818)	22	1.3	30	1.2	52	1.2
*Chrysomya putoria* (Wiedemann, 1818)	-	-	1	<0.1	1	<0.1
*Cochliomyia macellaria* (Fabricius, 1775)	16	0.9	29	1.2	45	1.1
*Hemilucilia segmentaria* (Fabricius, 1805)	353	20.3	313	12.7	666	15.9
*Hemilucilia semidiaphana* (Rondani, 1850)	111	6.4	172	7.0	283	6.7
*Hemilucilia benoisti* (Séguy, 1925)	1	<0.1	3	0.1	4	<0.1
*Paralucilia nigrofacialis* (Mello, 1969)	2	0.1	-	-	2	<0.1
Mesembrinellidae						
Mesembrinellinae						
*Mesembrinella bellardiana* (Aldrich, 1922)	69	4.0	154	6.3	223	5.3
*Mesembrinella peregrina* (Aldrich, 1922)	1	<0.1	4	0.2	5	<0.1
*Mesembrinella semihyalina* (Mello, 1967)	2	0.1	8	0.3	10	0.2
*Mesembrinella currani* (Guimarães, 1977)	4	0.2	1	<0.1	5	0.1
*Mesembrinella quadrilineata* (Fabricius, 1805)	1	<0.1	11	0.5	12	0.3
*Mesembrinella cyaneicyncta* (Surcouf, 1919)	-	-	2	<0.1	2	<0.1
*Mesembrinella randa* (Walker, 1849)	1	<0.1	3	0.1	4	0.1
*Mesembrinella benoisti* (Séguy, 1925)	-	-	1	<0.1	1	<0.1
*Mesembrinella aeneiventris* (Wiedemann, 1830)	1	<0.1	5	0.2	6	0.1
*Mesembrinella purpurata* (Aldrich, 1922)	2	0.1	3	0.1	5	0.1
Laneellinae						
*Laneella nigripes* (Guimarães, 1977)	93	5.3	201	8.2	294	7.0
Total	1739	100	2463	100	4202	100

**Table 2:** Similarity of Calliphoridae and Mesembrinellidae communities captured in three environments in the state of Rio de Janeiro: Forest: Jequitibá Nucleus of Três Picos State Park, municipality of Cachoeiras de Macacu; Rural: Cattle farm of the Federal Rural University of Rio de Janeiro, municipality of Seropédica; and Urban: Institute of Biosciences of the Federal University of the State of Rio de Janeiro, Urca Campus, municipality of Rio de Janeiro, between August 2021 and May 2023.

Environment	Forest	Rural	Urban
Forest	16	2	3
Rural	0.174	7	4
Urban	0.286	0.667	5

Lower triangle: Sorensen’s Similarity Index; Diagonal (gray): environmental richness; Upper triangle: Number of species common to both environments.

**Table 3:** Absolute (n) and relative (%) abundance of species of the Calliphoridae and Mesembrinellidae families captured in three distinct areas of the state of Rio de Janeiro: Forest: Jequitibá Nucleus of the Três Picos State Park, municipality of Cachoeiras de Macacu; Rural: Cattle farm of the Federal Rural University of Rio de Janeiro, municipality of Seropédica; and Urban: Institute of Biosciences of the Federal University of the State of Rio de Janeiro, Urca Campus, municipality of Rio de Janeiro, between August 2021 and May 2023.

Taxa	Forest		Rural		Urban		Total	
	n	%	n	%	n	%	n	%
Calliphoridae								
Luciliinae								
*Lucilia eximia*	2257	64.5	105	40.2	162	34.8	2524	58.9
*Lucilia cuprina*	-	-	20	7.7	-	-	20	0.5
Chrysomyinae								
*Chrysomya megacephala*	-	-	34	13.0	27	0.6	61	1.4
*Chrysomya albiceps*	-	-	49	18.8	3	0.1	52	1.2
*Chrysomya putoria*	-	-	1	<0.1	-	-	1	<0.1
*Cochliomyia macellaria*	-	-	45	17.2	-	-	45	1.1
*Hemilucilia segmentaria*	435	12.4	7	0.3	227	48.7	669	15.6
*Hemilucilia semidiaphana*	236	6.7	-	-	47	10.1	283	6.6
*Hemilucilia benoisti*	4	0.1	-	-	-	-	4	<0.1
*Paralucilia nigrofacialis*	2	0.1	-	-	-	-	2	<0.1
Mesembrinellidae								
Mesembrinellinae								
*Mesembrinella bellardiana*	223	6.4	-	-	-	-	223	5.2
*Mesembrinella peregrina*	5	0.1	-	-	-	-	5	<0.1
*Mesembrinella semihyalina*	10	0.3	-	-	-	-	10	0.2
*Mesembrinella currani*	5	0.1	-	-	-	-	5	<0.1
*Mesembrinella quadrilineata*	12	0.3	-	-	-	-	12	0.3
*Mesembrinella cyaneicyncta*	2	0.1	-	-	-	-	2	<0.1
*Mesembrinella randa*	4	0.1	-	-	-	-	4	<0.1
*Mesembrinella benoisti*	1	<0.1	-	-	-	-	1	<0.1
*Mesembrinella aeneiventris*	6	0.2	-	-	-	-	6	<0.1
*Mesembrinella purpurata*	5	0.1	-	-	-	-	5	<0.1
Laneellinae								
*Laneella nigripes*	294	8.4	-	-	-	-	294	6.9
Total	3501	100	261	100	466	100	4228	100

**Table 4:** Species of the Calliphoridae and Mesembrinellidae families potentially used as bioindicators/biomonitors captured in three environments in the state of Rio de Janeiro: Forest: Jequitibá Center of the Três Picos State Park, municipality of Cachoeiras de Macacu; Rural: Cattle farming at the Federal Rural University of Rio de Janeiro, municipality of Seropédica; and Urban: Institute of Biosciences of the Federal University of the State of Rio de Janeiro, Urca Campus, municipality of Rio de Janeiro, between August 2021 and May 2023.

Environment	Species	Stat	*p*-Value
Forest	*Laneella nigripes*	0.968	0.001
	*Mesembrinella bellardiana*	0.968	0.001
	*Lucilia eximia*	0.946	0.001
	*Mesembrinella quadrilineata*	0.559	0.011
	*Hemilucilia segmentaria*	0.727	0.018
	*Mesembrinella randa*	0.500	0.020
	*Mesembrinella semihyalina*	0.500	0.027
	*Mesembrinella currani*	0.500	0.030
Rural	*Cochliomyia macellaria*	0.707	0.001
	*Lucilia cuprina*	0.661	0.002
	*Chrysomya albiceps*	0.543	0.019
	*Chrysomya megacephala*	0.590	0.036
Urban	*-*	-	-

## Data Availability

All data is made available in the Open Science Framework, available online: https://doi.org/10.17605/OSF.IO/GWVNT.

## Abbreviations

The following abbreviations are used in this manuscript:

UNIRIO	Universidade Federal do Estado do Rio de Janeiro
UFRRJ	Universidade Federal Rural do Rio de Janeiro
